# Wireless Self‐Powered Optogenetic System for Long‐Term Cardiac Neuromodulation to Improve Post‐MI Cardiac Remodeling and Malignant Arrhythmia

**DOI:** 10.1002/advs.202205551

**Published:** 2023-01-25

**Authors:** Liping Zhou, Yuanzheng Zhang, Gang Cao, Chi Zhang, Chen Zheng, Guannan Meng, Yanqiu Lai, Zhen Zhou, Zhihao Liu, Zihan Liu, Fuding Guo, Xin Dong, Zhizhuo Liang, Yueyi Wang, Shishang Guo, Xiaoya Zhou, Hong Jiang, Lilei Yu

**Affiliations:** ^1^ Department of Cardiology Renmin Hospital of Wuhan University Hubei Key Laboratory of Autonomic Nervous System Modulation Cardiac Autonomic Nervous System Research Center of Wuhan University Taikang Center for Life and Medical Sciences Wuhan University Cardiovascular Research Institute Wuhan University Hubei Key Laboratory of Cardiology Wuhan 430060 P. R. China; ^2^ Hubei Yangtze Memory Laboratories Key Laboratory of Artificial Micro, and Nano‐structures of Ministry of Education School of Physics and Technology Wuhan University Wuhan 430072 P. R. China; ^3^ Biomedical Center College of Veterinary Medicine Huazhong Agricultural University Wuhan 430072 P. R. China; ^4^ Wuhan National Laboratory for Optoelectronics Huazhong University of Science and Technology Wuhan 430072 P. R. China

**Keywords:** cardiac remodeling, neuromodulation, triboelectric nanogenerator, ventricular arrhythmia, wireless self‐powered optogenetics

## Abstract

Autonomic imbalance is an important characteristic of patients after myocardial infarction (MI) and adversely contributes to post‐MI cardiac remodeling and ventricular arrhythmias (VAs). A previous study proved that optogenetic modulation could precisely inhibit cardiac sympathetic hyperactivity and prevent acute ischemia‐induced VAs. Here, a wireless self‐powered optogenetic modulation system is introduced, which achieves long‐term precise cardiac neuromodulation in ambulatory canines. The wireless self‐powered optical system based on a triboelectric nanogenerator is powered by energy harvested from body motion and realized the effective optical illumination that is required for optogenetic neuromodulation (ON). It is further demonstrated that long‐term ON significantly mitigates MI‐induced sympathetic remodeling and hyperactivity, and improves a variety of clinically relevant outcomes such as improves ventricular dysfunction, reduces infarct size, increases electrophysiological stability, and reduces susceptibility to VAs. These novel insights suggest that wireless ON holds translational potential for the clinical treatment of arrhythmia and other cardiovascular diseases related to sympathetic hyperactivity. Moreover, this innovative self‐powered optical system may provide an opportunity to develop implantable/wearable and self‐controllable devices for long‐term optogenetic therapy.

## Graphical abstract

In the present study, a wireless self‐powered optogenetic modulation system is introduced, which achieves long‐term precise cardiac neuromodulation in ambulatory canines. Long‐term optogenetic neuromodulation significantly improves MI‐induced cardiac autonomic, electrical, functional, and structural remodeling, and thus, suppresses post‐MI ventricular arrhythmogenesis.

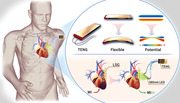

## 1 Introduction

Autonomic imbalance, manifested by increased sympathetic and decreased parasympathetic nerve activity, is an important characteristic of patients after myocardial infarction (MI) and adversely contributes to post‐MI cardiac remodeling and ventricular arrhythmias (VAs).^[^ ^1^, ^2^, ^3^ ^]^ Neuromodulation strategies that directly target the sympathetic nervous system, such as stellectomy or stellate ganglion blockade, have been utilized to improve post‐MI cardiac remodeling and VAs both in experimental and clinical studies.^[^ ^4^, ^5^, ^6^, ^7^ ^]^ Thus, strategical optimization to enhance the precision and controllability of cardiac neuromodulation is of great significance for promoting clinical application.

Optogenetics is a novel powerful neuromodulatory technology that combines optics and genetics to achieve spatiotemporal precise activation or inhibition of targeted neurons.^[^ ^8^, ^9^, ^10^ ^]^ Owing to its great advantages over conventional neuromodulatory approaches in cell‐specific control without systemic pharmacologic agents or causing permanent nerve damage, optogenetics has been innovatively applied for peripheral nerve control for targeted organs,^[^ ^11^ ^]^ such as the heart,^[^ ^12^, ^13^ ^]^ lung,^[^ ^14^, ^15^ ^]^ bladder,^[^ ^16^ ^]^ and gastrointestinal system,^[^ ^17^ ^]^ to modulate organ function. Our previous study has demonstrated that optogenetic modulation of cardiac sympathetic neurons in the left stellate ganglion (LSG) could precisely and reversibly inhibit cardiac sympathetic hyperactivity, thereby increasing ventricular electrophysiological stability and suppressing acute ischemia‐induced VAs.^[^ ^18^ ^]^ However, optogenetic cardiac neuromodulation is currently limited to short‐term studies using wired optical devices in in vitro perfused hearts or anesthetized animals.^[^ ^12^, ^19^, ^20^, ^21^, ^22^ ^]^ Tethered operation modules or wired energy supply of the optical devices have hindered the application of long‐term optogenetic cardiac neuromodulation in awake freely moving animals and further clinical translation.

Recent advances in wireless optogenetic technology have overcome this limitation by developing implantable wireless optical devices. Wireless optogenetic devices with soft and flexible light‐emitting diode (LED) probes have been developed for in vivo optogenetic control of the central and peripheral nervous system in freely moving animals.^[^ ^23^, ^24^, ^25^, ^26^ ^]^ However, batteries for supplying power to wireless devices have limited storage capacity and lifetime, and also increased weight and size of the devices, which may cause inconveniences during implantation and operation. The development of wireless and battery‐free power supply technologies has provided effective approaches to enable devices with miniaturized size, lightweight, and unlimited operation lifetime. The wireless and battery‐free power supply technologies include internal power harvesting and external power transmission.^[^ ^27^ ^]^ The advanced technology of nanogenerators has provided a great chance to convert biomechanical energy in the human body into electricity for powering biomedical systems for cardiac pacing,^[^ ^28^, ^29^ ^]^ neuromodulation,^[^ ^30^, ^31^ ^]^ or optical therapy.^[^ ^32^, ^33^ ^]^ Inspired by these advances, we developed a wireless self‐powered optogenetic system suitable for wireless cardiac sympathetic modulation in awake freely moving canines. The wireless self‐powered optical system based on a triboelectric nanogenerator (TENG) is powered by energy harvested from body motion and realized effective optical illumination that is required for optogenetic neuromodulation (ON). Moreover, we developed an implantable, battery‐free wireless optical system with a magnetic resonant coupling external power transmission module that can provide a reliable and constant power supply for long‐term in vivo optogenetic neuromodulation. The implantable, battery‐free wireless optogenetic system combines a soft, flexible LED opto‐electrode implanted onto LSG to directly illuminate the LSG for optogenetic modulation, and a power receiver implanted under the skin to harvest energy from an external wireless charging module to power the implanted LED system. This system provides a critical advantage over wired optical devices and enables long‐term wireless cardiac sympathetic modulation in awake freely moving canines. We further investigated whether long‐term precise cardiac neuromodulation via this wireless optogenetic system could safely and efficiently inhibit MI‐induced LSG hyperactivity and improve MI‐induced cardiac remodeling and arrhythmogenesis in ambulatory post‐MI canines.

## 2 Results

### 2.1 Overview of the Self‐Powered Optical System

**Figure 1:**
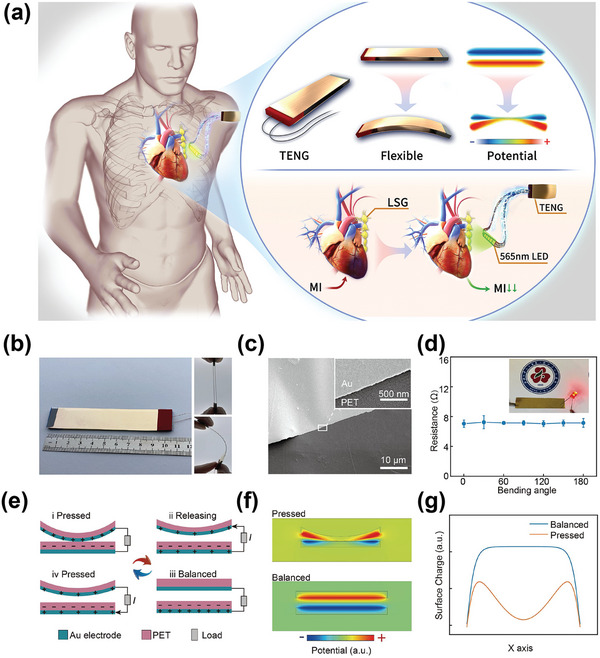
Overview of the self‐powered optical system. The self‐powered optical system consists of a TENG‐based energy harvesting unit, a power manage unit (PMU), and LED opto‐electrode. a) The optogenetic neuromodulation for cardioprotection by the self‐powered optical system is presented. b) Optical image of the TENG. The inset indicates this TENG is flexible and can be bent by a finger. c) SEM image of the Au/PET. d) Resistance of the electrode under different bending angles. e) Schematics diagrams of the working mechanism of TENG. f) FEA simulation results of TENG under pressed and balanced state. g) Corresponding surface charge distribution curves along the surface of the electrode.

The self‐powered optical system consists of a TENG‐based energy harvesting unit, a power manage unit (PMU), and an LED opto‐electrode (**Figure** **1**a). The TENG converts biomechanical energy from body motion into electrical energy. The optogenetic neuromodulation for cardioprotection by the self‐powered optical system is also presented. Figure 1b presents the optical image of the TENG. The inset of Figure 1b indicates this device can be bent by a finger, proving good flexibility. The SEM image of Au/PET is given in Figure 1c. It can be seen that the Au film is uniformly and densely deposited on the PET surface, so this flexible electrode still has good conductivity in the bent state. The resistance change results of the electrode under different bending angles are plotted in Figure 1d, proving that the flexible electrode is sufficient for use in almost all bending scenarios, which is crucial for harvesting biomechanical energy from daily high‐freedom movements. It is well known that PET is widely used as a friction material due to its excellent flexibility and high electronegativity.^[^ ^34^, ^35^ ^]^ It is worth noting that Au is the friction material, as well as the electrode in this work. The structure schematic and detailed working principle are shown in Figure 1e. This TENG works in the vertical contact‐separation mode, and the work process can be divided into four stages. In the pressed state (Figure 1ei), the charge transfers from the Au to the PET once the PET contacts the Au. Meanwhile, the electrode is positively charged owing to the triboelectrification effect. When the external force is released, according to electrostatic induction, the positive charge in the top electrode is transferred to the bottom electrode (Figure 1eii) until a balanced state (Figure 1eiii). When the device receives external compression again, all positive charges will migrate from the bottom to the top electrode (Figure 1eiv).^[^ ^36^, ^37^ ^]^ In summary, an alternating electrical output can be generated by continuous pressure on the TENG. Finite element analysis (FEA) based on COMSOL is utilized to examine the working principle, as revealed in Figure 1f. We simulated the potential distribution of the TENG in the compressed and balanced states and performed numerical statistics of charge distribution in the lower electrode under different states (Figure 1g). From figure 1g, the amount of induced charge in the lower electrode in the pressed state is higher than that of the balanced state, which agrees well with the analysis mentioned in Figure 1f.

### 2.2 Characterization of the TENG and Output Performance of the PENG‐Based Optical System

**Figure 2:**
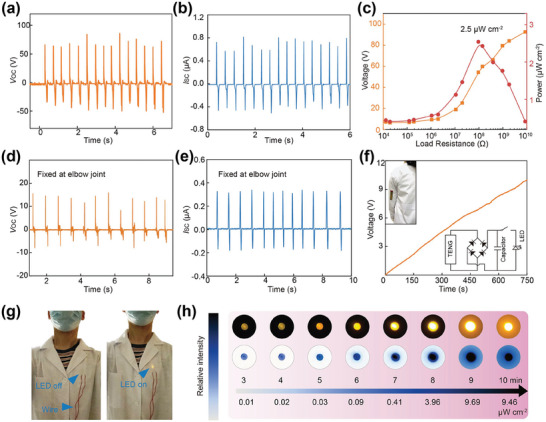
Characterization of the TENG and output performance of the PENG‐based optical system. a) *V*_OC_ and b) *I*_SC_ of the TENG. c) Dependence of the output voltage and instantaneous power density at different external load resistances. d) Output voltage and e) current of PENG fixed at the elbow joint. f) Charging curves of a 2.2 µF capacitor charged by TENG. g) LED lighted by the wearable self‐powered optogenetic modulation system. h) Light intensity of the LED changing with charging time.

To study the output characteristics of the TENG in detail, we temporarily use a homemade linear motor to simulate body motion. The open‐circuit voltage (*V*_OC_) and short‐circuit current (*I*_SC_) of the TENG were measured, which can reach about 78 V and 0.7 µA, as presented in **Figure** **2**a,b. It should be noted that, as for flexible nanogenerators, asymmetrical positive and negative peaks can be comprehended by the fact that the pressing process is exerted by the external force, whereas the releasing process is predominated by the resilience of the device itself.^[^ ^38^ ^]^ Therefore, the motion behavior of the device in the compression and release stages is slightly different, resulting in a slight deviation of the output signal. Additionally, after each release process, a small tensile strain will be produced due to the good elasticity of the PET, which can change the electric field between the PET and Au electrode, thus emerging a weak electrical output signal.^[^ ^39^ ^]^ We also measure the electrical performance of TENG under different external load resistances, as plotted in Figure 2c and Figure S1, Supporting Information. The output power density (*P*) of TENG is calculated by the equation as follows

(1) $$P=\frac{UI}{S}$$

where *U*, *I*, and *S* are the output voltage, current, and effective area of the TENG. The maximum *P* can reach 2.5 µW cm^−2^ at a load resistance of about 100 MΩ. To evaluate the feasibility of this wearable self‐powered optical system, we fixed the TENG on the human elbow joint and tested electrical output, as shown in Figure 2d,e. Binding with the elbow joint, the output voltage and current of the TENG are about 15 V and 0.3 µA, respectively. Furthermore, a 2.2 µF capacitor was connected to TENG through a rectifier bridge to store the mechanical energy generated by the bent elbow joint, and the corresponding charging curve is recorded in Figure 2f. The inset of Figure 2f shows the circuit diagram and experimental image of the TENG‐LED optical system. When the elbow is continuously bent for 10 min, the voltage of the wearable self‐powered optical system can reach 10 V. Once the switch is closed, the LED will be illuminated, as displayed in Figure 2g. The luminous intensity of the LED is related to the operating time of the TENG (Figure 2h), and the LED luminous intensity reaches 9.46 µW cm^−2^ after 10 min mechanical energy harvesting by TENG. The results showed that the longer the TENG operates, the more electrical energy is captured and stored, which indicates the feasibility of the TENG‐LED self‐powered optical system for long‐term optogenetic neuromodulation.

### 2.3 Wireless LED Device Implantation, Location Verification, and Heat Generation Test

**Figure 3:**
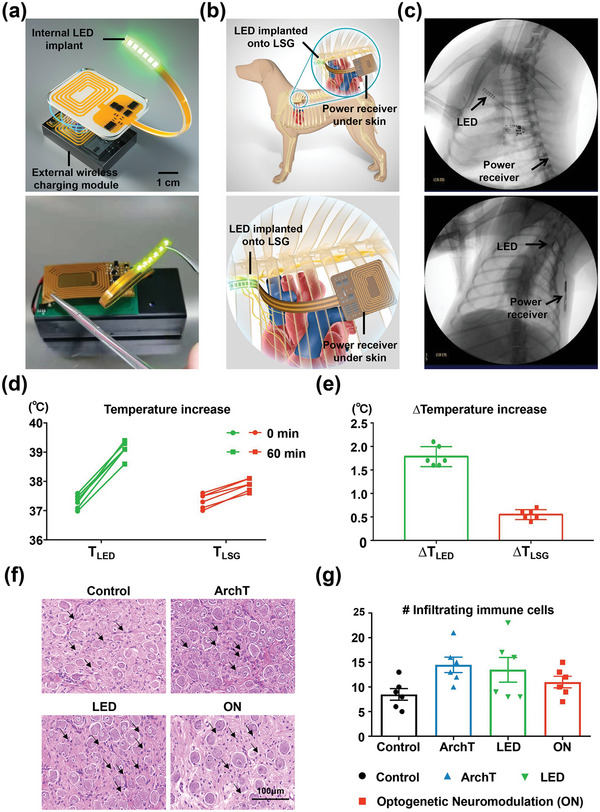
Wireless implantable optogenetic system design and implantation diagram, device location examination and heat generation test, and LSG neuronal health. a) Schematic diagram and optical image of the implantable, battery‐free wireless optogenetic system. b) Diagram of the wireless LED device implantation in canines. c) Chest X‐ray examination confirmed that the LED implant was maintained at the corresponding position. d,e) Thermal property measurement on the surface of LED and the surface of the LSG during 1 h illumination. f,g) Representative hematoxylin and eosin images and quantification of infiltrating immune cells from the LSG. The black arrowheads in (f) represent infiltrating immune cells. Scale bar in (f) = 100 µm; Data are mean ± SEM. Statistical analysis was performed using one‐way analysis of variance (ANOVA) (g). Only statistically significant differences are indicated (*n* = 6 per group).

To verify the cardio‐protective effect of long‐term optogenetic neuromodulation in awake freely moving canines, the wireless implantable optical system requires a reliable and constant power supply and suitable illumination parameters (20 Hz frequency, 40% duty cycle, 20 ms pulse width) to enable effective optogenetic operation. Thus, we proposed a compromise combining a magnetic resonant coupling external power transmission module to supply the wireless LED opto‐electrode. This wireless implantable optogenetic neuromodulation system consists of an internal LED implant and an external wireless charging module (**Figure** **3**a). The optical images and detailed circuit design diagram of the LED system, as well as the movie of LED working mode, are presented (Figure S2 and Video S1, Supporting Information). The internal LED opto‐electrode was implanted onto the LSG, and the power receiver that connects with the LED was implanted subcutaneously in the chest, about 1 cm deep under the skin, to extract radio‐frequency (RF) energy through coupling with the external wireless charging module tied to the corresponding position on the chest wall (Figure 3b). After 4 weeks, a chest X‐ray examination confirmed that the location of the implanted LED devices was maintained at the corresponding position (Figure 3c).

Heat generation from the LED during illumination is a critical concern as a significant increase in tissue temperature could lead to alterations of neural activity and damage to surrounding tissue.^[^ ^40^ ^]^ We characterized the thermal property of the implanted LED device in vivo using an optical fiber temperature sensor system. The fiber‐optics sensor was placed on the surface of the LED units, together inside the encapsulated soft silicone rubber sheet coating, and then implanted onto the surface of the LSG, to measure the thermal effect of the LED illumination. Figure S3a, Supporting Information, shows the temperature‐to‐time curve at different LED operation conditions (illumination parameters: 20 Hz, 40% duty cycle, 20 ms pulse width, 5 mW mm^−2^). Continuous LED illumination caused a temperature increase from 37.3 to 39.3 °C of LED and then reached a stable level. When the LED was turned off, the temperature fell quickly to the baseline (37.4 °C). Heat generation test during 1 h LED illumination was performed six times and the results showed that the temperature measured on the surface of the LED units increased by 1.78 ± 0.21 °C during 1 h illumination (Figure 3d,e and Figure S3b, Supporting Information). In view of the insulation property of the encapsulation, we also measured the temperature on the surface of LSG, which is outside of the encapsulated soft silicone rubber sheet coating, to detect the thermal response on the LSG tissue. The results showed that the temperature measured on the surface of the LSG tissue increased by 0.55 ± 0.10 °C (Figure 3d,e and Figure S3b, Supporting Information). Results indicate that the LED illumination in vivo only induces a limited temperature increase of LSG tissue.

LSG nerve tissue and systemic reaction to the LED device implantation and illumination were assessed. At the end of the experiment, LSG and surrounding tissue were excised to examine the nerve inflammatory reaction. The immune cells were identified according to the cell morphology in HE staining images including neutrophil and macrophage. HE staining images of LSG from four groups were presented and infiltrating immune cells were counted (Figure 3f,g). The result demonstrated that the 4‐week LED implantation and illumination did not induce significant changes in immune cell infiltration among the four groups. Moreover, we measured serum inflammatory markers, including IL‐1*β*, IL‐6, and TNF‐*α*, at baseline and 4 weeks after optogenetic modulation. The results showed that no obvious significance was observed of IL‐1*β*, IL‐6, and TNF‐*α* concentration among four groups, both at baseline and 4 weeks after optogenetic modulation (Figure S4, Supporting Information). The results proved that LED device implantation or long‐term optogenetic modulation did not induce excessive LSG nerve tissue or systemic inflammatory response, indicating the LED device is biocompatible and safe for long‐term implantation and neuromodulation in canines.

### 2.4 Long‐Term Wireless Optogenetic Modulation Inhibited MI‐Induced LSG Neuronal Remodeling

**Figure 4:**
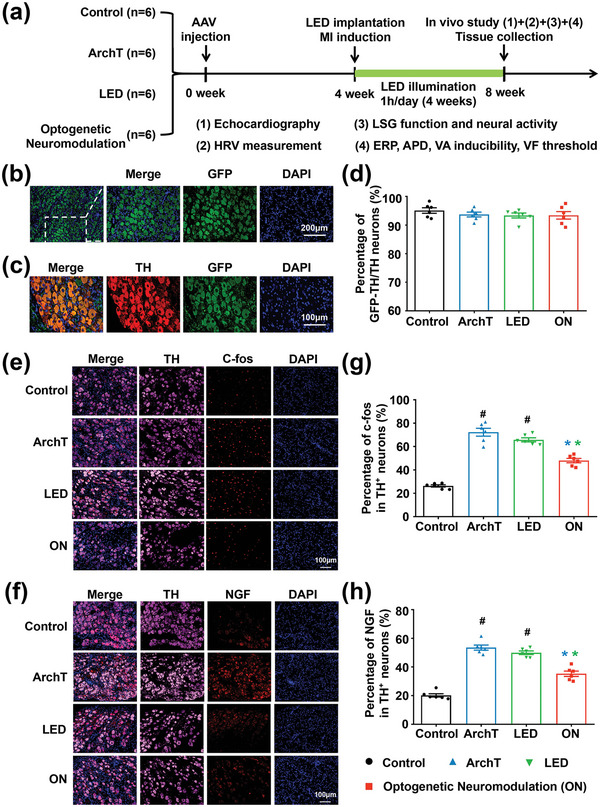
Long‐term wireless optogenetic modulation inhibited MI‐induced LSG neuronal remodeling. a) Experimental protocol flow. b–d) Immunofluorescent staining and quantification analysis verified that GFP/GFP‐ArchT was successfully and extensively expressed in LSG sympathetic neurons in four groups. Representative images of c‐fos (e, red) and NGF (f, red) expression in LSG TH+ (e,f, pink) sympathetic neurons in four groups. Long‐term wireless optogenetic neuromodulation suppressed MI‐induced LSG neuronal remodeling with reduced c‐fos and NGF expression in TH+ neurons (g,h). NGF, nerve growth factor. Scale bar in (b) = 200 µm; Scale bar in (c,e,f) = 100 µm. Data are mean ± SEM. ^#^ *p* < 0.05 versus the control group and *p < 0.05 versus ArchT/LED group by one‐way ANOVA with Tukey's comparisons test (*n* = 6 per group).

The study protocol is outlined in **Figure** **4**a. Twenty‐four beagle canines were randomly divided into the control group (*n* = 6, GFP expression with sham MI), the ArchT group (*n* = 6, ArchTexpression with MI), the LED group (*n* = 6, GFP expression with MI and LED illumination), and the ON group (*n* = 6, ArchTexpression with MI and LED illumination). LSG neurons were targeted to express an inhibitory light‐sensitive protein ArchT and/or the reporter GFP via AAV micro‐injection in four groups. At the end of the experiment, LSGs were harvested for histological examination. LSG slices stained with anti‐GFP showed that GFP/ArchT‐GFP was successfully and extensively expressed in LSG neurons (Figure 4b). Double immunofluorescence staining with anti‐TH and anti‐GFP revealed that 93.41 ± 1.32% of TH^+^ cells were GFP^+^ in the optogenetic neuromodulation group, 93.70 ± 0.85% in the ArchT group, and 93.36 ± 0.87% in the LED group (Figure 4c,d).

Figure 4e,f shows the representative images of double immunofluorescence staining with TH and c‐fos/nerve growth factor (NGF) of LSG tissue in four groups. Compared to the control group, MI resulted in significant increases in c‐fos and NGF expression in TH^+^ neurons in both the ArchT group and the LED group, which were attenuated by wireless optogenetic neuromodulation (Figure 4g,h). However, studies have shown that MI could induce significant and persistent upregulation of NGF in LSG for months, resulting in sympathetic hyperactivity and hyperinnervation.^[^ ^41^, ^42^ ^]^ Periodic optogenetic neuromodulation could only partially attenuate but not completely reverse the upregulation of NGF. Notably, no significant difference was observed in the MI‐induced cardiac neuronal remodeling between the ArchT group and the LED group, indicating that LED illumination alone did not affect the LSG neural activity. Moreover, we analyzed the HE staining images of LSG tissue from four groups and measured the LSG neuron size. The results showed that 4‐week MI induced morphological changes with an increase in LSG neuron diameter in the ArchT group and LED group, which was inhibited by long‐term optogenetic modulation (Figure S5, Supporting Information).

### 2.5 Long‐Term Wireless Optogenetic Modulation Inhibited MI‐Induced LSG Hyperactivity and Systemic ANS Imbalance

**Figure 5:**
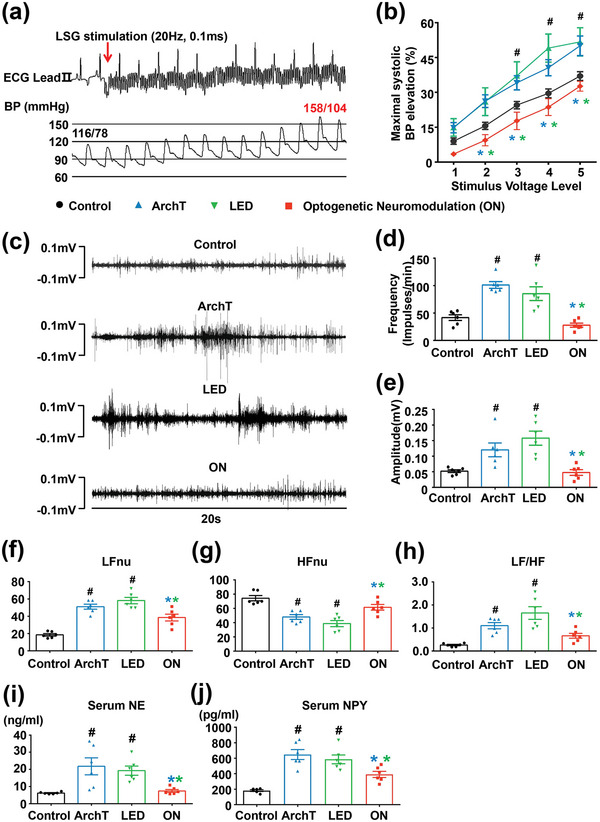
Long‐term wireless optogenetic modulation inhibited MI‐induced LSG hyperactivity and systemic ANS imbalance. a) Representative examples of BP elevation in response to LSG stimulation. b) Long‐term wireless optogenetic modulation significantly inhibited LSG function. The *x*‐axis numbers 1–5 represent five incremental high‐frequency stimulus voltage levels (level 1 = 1 to 4 V; level 2 = 5 to 7 V; level 3 = 7.5 to 10 V; level 4 = 10 to12.5 V; level 5 = 12.5‐15 V). c) Representative images of LSG neural activity of four groups. Long‐term wireless optogenetic modulation inhibited MI‐induced LSG hyperactivity, with both decreased d) frequency and e) amplitude of LSG neural firing. Long‐term wireless optogenetic inhibition of cardiac sympathetic nerve activity significantly improved HRV, attenuated MI‐induced increase in f) LFnu, h) LF/HF ratio, and g) decrease in HFnu. i,j) Long‐term wireless optogenetic inhibition of cardiac sympathetic nerve activity significantly reduced plasma norepinephrine and neuropeptide Y concentration. HFnu, normalized unit of high frequency; LFnu, normalized unit of low frequency; LF/HF, the ratio between LF and HF; NE, norepinephrine; NPY, neuropeptide Y. Data are mean ± SEM. ^#^ *p* < 0.05 versus the control group and *p < 0.05 versus ArchT/LED group by one‐way ANOVA or two‐way repeated‐measures ANOVA with Tukey's multiple comparisons test (*n* = 6 per group).

Longitudinal changes in blood pressure (BP) and heart rate (HR) during the viral transfection and optogenetic modulation were evaluated. No significant changes in the systolic and diastolic BP or the HR were observed among the four groups (Figure S6a–c, Supporting Information), indicating that optogenetic cardiac neuromodulation did not affect rest BP and HR of canines. The maximal systolic BP change in response to LSG electrical stimulation was measured to assess the LSG function (**Figure** **5**a). Compared to the control group, MI induced a significant increase in LSG function in both the ArchT group and the LED group. However, long‐term wireless optogenetic neuromodulation significantly inhibited MI‐induced increase in LSG function (Figure 5b). Moreover, neural activity recording (Figure 5c) revealed that MI significantly increased the frequency and amplitude of LSG neural activity in both the ArchT group and the LED group, which was significantly inhibited by long‐term wireless optogenetic neuromodulation (Figure 5d,e). No significant difference was observed in the MI‐induced cardiac sympathetic hyperactivity or neuronal remodeling between the ArchT group and LED group, indicating that LED illumination alone did not affect the LSG neural activity. These results indicate that long‐term optogenetic neuromodulation significantly suppressed MI‐induced LSG hyperactivity and neuronal remodeling.

We also evaluated whether long‐term wireless optogenetic inhibition of LSG hyperactivity could mitigate the MI‐induced autonomic nervous system (ANS) imbalance. HRV has been widely used as an index of the ANS function. The power spectral variables, including the low frequency (LF) and high frequency (HF) components, were analyzed and the normalized unit of LF and HF (LFnu and HFnu) and the ratio between LF and HF (LF/HF) were calculated. MI induced a significant increase in LFnu and LF/HF, and a decrease in HFnu as compared to the control group (Figure 5f–h). However, long‐term wireless optogenetic inhibition of LSG nerve hyperactivity significantly attenuated the MI‐induced increase in LFnu and LF/HF, and the decrease in HFnu as compared to both the ArchT group and the LED group (both *p* < 0.05) (Figure 5f–h). In addition, the plasma concentration of cardiac sympathetic neurotransmitter norepinephrine (NE) and co‐transmitter neuropeptide Y (NPY) were increased in the ArchT and LED group but was decreased by long‐term wireless optogenetic neuromodulation (Figure 5i,j). These results indicate that long‐term wireless optogenetic modulation of cardiac sympathetic hyperactivity could improve MI‐induced ANS imbalance.

### 2.6 Long‐Term Optogenetic Neuromodulation Improved MI‐Induced LV Dysfunction

**Figure 6:**
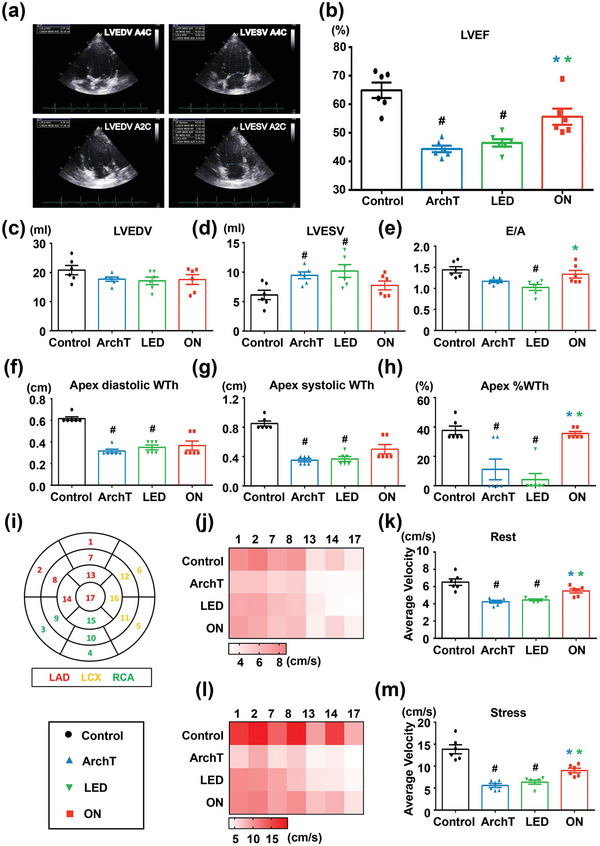
Long‐term optogenetic neuromodulation improved MI‐induced LV dysfunction and remodeling. a) Schematic diagram of cardiac four‐chamber and two‐chamber view for volume calculations using biplane Simpson's method. Long‐term wireless optogenetic cardiac sympathetic inhibition improved cardiac function with b) increased LVEF compared with ArchT/LED group and e) increased E/A compared with LED group. There were no significant differences between the optogenetic neuromodulation group and ArchT/LED group in c) LVEDV and d) LVESV. f–h) Long‐term wireless optogenetic cardiac sympathetic inhibition attenuated LV segmental wall thinning with increased % systolic wall thickening at the apex. i) Display of the 17 myocardial segments of the left ventricle. Long‐term wireless optogenetic cardiac sympathetic inhibition increased peak systolic velocity of the LAD‐perfused segments both j,k) at rest and l,m) at dobutamine stress. LVEF, left ventricular ejection fraction; LVEDV, left ventricular end‐diastolic volume; LVESV, left ventricular end‐systolic volume; WTh, wall thickness; LAD, left anterior descending artery; LCX, left circumflex artery; RCA,= right coronary artery. Data are mean ± SEM. ^#^ *p* < 0.05 versus the control group and *p < 0.05 versus ArchT/LED group by one‐way ANOVA or two‐way repeated‐measures ANOVA with Tukey's multiple comparisons test (*n* = 6 per group).

We then investigated whether long‐term wireless optogenetic inhibition of LSG hyperactivity could improve MI‐induced left ventricular (LV) dysfunction. Representative apical four‐chamber and two‐chamber views for volume calculations using biplane Simpson's method are shown in **Figure** **6**a. Results showed that MI caused significant LV dysfunction with increased left ventricular end‐systolic volume (LVESV), reduced left ventricular ejection fraction (LVEF), and decreased E/A (Figure 6b–e) compared to the control group. However, long‐term optogenetic inhibition of LSG hyperactivity significantly improved LVEF as compared to the MI group (*p* < 0.05) (Figure 6b). MI also resulted in LV segmental wall thinning as reflected by reduced diastolic and systolic wall thickness, as well as % systolic wall thickening at the apex (Figure 6f–h), while the % systolic wall thickening was improved by long‐term optogenetic neuromodulation (Figure 6h). Importantly, long‐term wireless optogenetic neuromodulation of LSG hyperactivity attenuated MI‐induced LV regional wall‐motion abnormality and improved myocardial functional reserve, which was manifested by increased LV segmental peak systolic velocity of the left anterior descending artery (LAD)‐perfused segments both at rest and at dobutamine stress as compared to the two sham MI groups (Figure 6i–m).

### 2.7 Long‐Term Optogenetic Neuromodulation Suppressed LV Structural Remodeling

**Figure 7:**
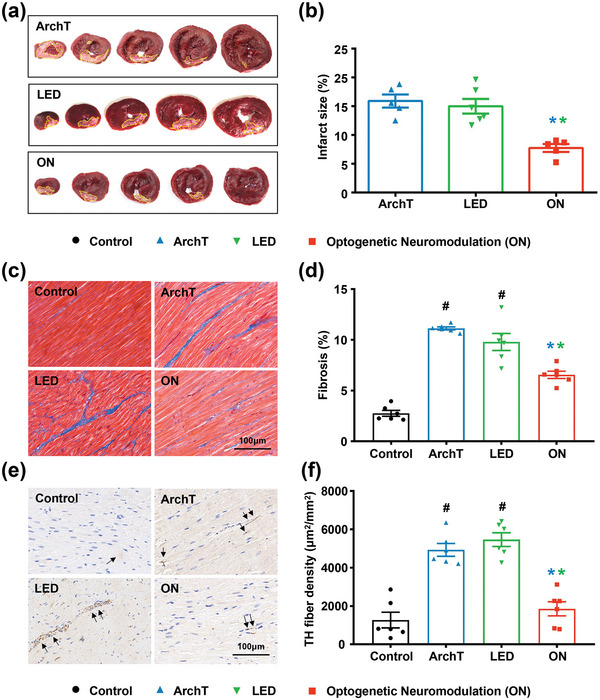
Long‐term optogenetic neuromodulation improved LV structural remodeling. a) Representative images of LV infarct size in the MI group and the optogenetics group. b) Long‐term wireless optogenetic cardiac sympathetic inhibition significantly reduced LV infarct size, c,d) suppressed LV fibrosis, and e,f) fiber innervation. The black arrowheads in (e) represent TH^+^ sympathetic fibers. Data are mean ±SEM. ^#^ *p* < 0.05 versus the control group and *p < 0.05 versus ArchT/LED group by one‐way ANOVA with Tukey's multiple comparisons test (*n* = 6 per group).

We further investigated the effect of long‐term wireless optogenetic modulation on post‐MI structural remodeling. Infarct size was assessed with TTC staining and representative MI images of three groups are shown in **Figure** **7**a. All the MI staining images and MI size quantification from the ArchT group, the LED group, and the optogenetic neuromodulation group are presented in Figure S7a–c, Supporting Information. Results showed that long‐term wireless optogenetic cardiac sympathetic inhibition reduced myocardial infarction size compared to two sham MI groups (Figure 7b). Moreover, Masson's trichrome staining was performed to assess interstitial fibrosis in the non‐infarcted myocardium of the LV base (Figure 7c). MI induced a significant increase in myocardial interstitial fibrosis (ArchT group: 11.12 ± 0.15% vs 2.76 ± 0.29%, *p* < 0.05 vs control group; LED group: 9.79 ± 0.84% vs 2.76 ± 0.29%, *p* < 0.05 vs control group), which was significantly reduced by long‐term optogenetic neuromodulation (6.55 ± 0.88%, *p* < 0.05) (Figure 7d). In addition, MI‐induced TH^+^ sympathetic fiber hyper‐innervation in LV non‐infarcted myocardium was also significantly suppressed by long‐term optogenetic neuromodulation (Figure 7e,f).

### 2.8 Long‐Term Optogenetic Neuromodulation Increased Ventricular Electrophysiological Stability and Reduced VA Susceptibility

**Figure 8:**
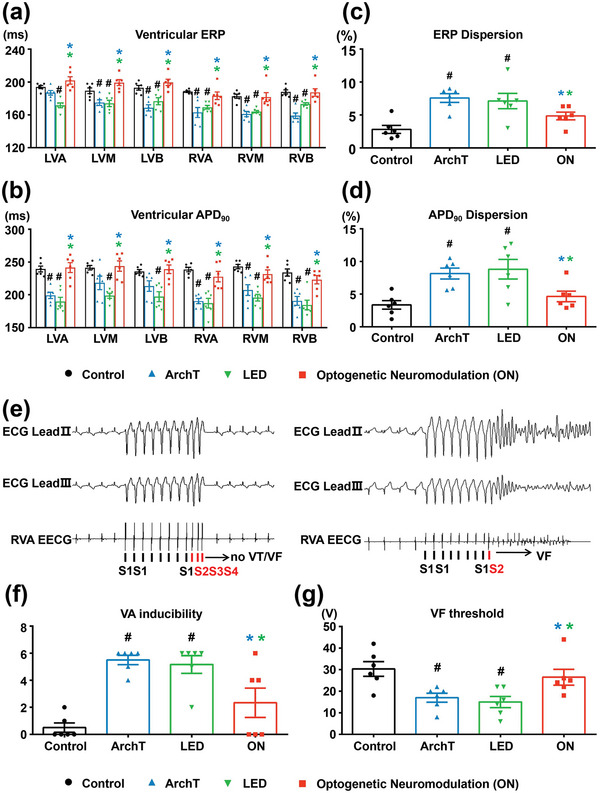
Long‐term optogenetic neuromodulation improved ventricular electrophysiological stability and reduced VA susceptibility. Long‐term wireless optogenetic modulation prolonged ventricular a) ERP and b) APD_90_ with c,d) reduced spatial dispersion. e) Typical images of ECG lead II, ECG lead III, and epicardium ECG (EECG) recorded from RVA when performing programmed electrical stimulation. Long‐term wireless optogenetic modulation significantly f) decreased VA inducibility and g) increased VF threshold. APD90 = 90% repolarization of APD. ERP, effective refractory period; LVA, LV apical peri‐infarct zone; LVM, LV median area; LVB, LV base; RVA, right ventricular apex; RVM, RV median area; RVB, RV base. Data are mean ± SEM. ^#^ *p* < 0.05 versus the control group and *p < 0.05 versus ArchT/LED group by one‐way ANOVA or two‐way repeated‐measures ANOVA with Tukey's multiple comparisons test (*n* = 6 per group).

Ventricular electrophysiological properties were assessed by programmed stimulation to measure the effective refractory period (ERP) and action potential duration (APD) at multiple ventricular sites (**Figure** **8**a,b and Figure S8, Supporting Information). Compared to the control group, MI significantly deteriorated ventricular electrophysiological stability, as reflected by significantly shortened ERP and 90% repolarization of APD (APD_90_) with increased spatial dispersion in both the ArchT group and the LED group. However, long‐term optogenetic inhibition of LSG neural activity significantly stabilized ventricular electrophysiological properties with prolonged ERP (Figure 8a) and APD_90_ (Figure 8b) and reduced spatial dispersion (Figure 8c,d) as compared to the two sham MI groups. In addition, we evaluated the effect of long‐term wireless optogenetic neuromodulation on VA susceptibility by programmed pacing to measure VA inducibility (Figure 8e) and by burst pacing to measure ventricular fibrillation (VF) threshold. MI significantly exacerbated VA susceptibility with increased VA inducibility and decreased VF threshold in both the ArchT group and the LED group. However, optogenetic neuromodulation significantly attenuated MI‐induced increase in VA inducibility (2.3 ± 1.1 vs 5.5 ± 0.3, *p* < 0.05 vs ArchT group; 2.3 ± 1.1 vs 5.2 ± 0.7, *p* < 0.05 vs LED group) (Figure 8f) and decrease in VF threshold (27 ± 4 V vs 17 ± 2 V, *p* < 0.05 vs ArchT group; 27 ± 4 V vs 15 ± 3 V, *p* < 0.05 vs LED group) (Figure 8g).

## 3 Discussion

### 3.1 Major Findings

In the present study, we developed a wireless self‐powered optical system based on a TENG that can harvest mechanical energy from body motion and convert it into electricity, and realize effective optical illumination that is required for optogenetic neuromodulation. Further, we achieved long‐term precise cardiac neuromodulation via the fully implantable, battery‐free wireless optogenetic system in ambulatory canines. Long‐term optogenetic neuromodulation significantly inhibited MI‐induced LSG hyperactivity and remodeling and improved a variety of clinically relevant outcomes. Overall, long‐term wireless optogenetic cardiac sympathetic modulation significantly improved MI‐induced autonomic, functional and structural, and electrophysiological remodeling, thereby suppressing post‐MI VA susceptibility.

### 3.2 Optogenetic Modulation of Cardiac Sympathetic Circuits: From Wired to Wireless

Optogenetics is a novel technology that enables spatiotemporal precise and reversible neural activation or inhibition of genetically modified neurons with light. Optogenetics has been applied for central and peripheral cardiac sympathetic neuromodulation, to uncover the neural mechanisms of cardiovascular diseases or explore their therapeutic potentials.^[^ ^9^, ^11^ ^]^ Our previous study has demonstrated that optogenetics could precisely and reversibly modulate cardiac sympathetic nerve activity, and thus, suppressing acute ischemia‐induced VAs.^[^ ^18^ ^]^

In addition, a series of previous studies have successfully employed optogenetic neuromodulation to precisely modulate the vagus nerve in both rodents^[^ ^43^, ^44^ ^]^ and large animals,^[^ ^45^ ^]^ achieving not only bidirectional modulation^[^ ^44^ ^]^ but also subpopulation‐selective modulation.^[^ ^45^ ^]^ These studies further elucidated the important role of the vagus nerve in diseases and the therapeutic potential of optogenetic vagal modulation, providing important contexts to the field of neuromodulation of cardiac function. However, due to tethered operation with wired optical devices, optogenetic cardiac neuromodulation is currently limited to short‐term studies in in vitro perfused hearts or anesthetized animals.^[^ ^13^, ^18^, ^19^, ^20^, ^21^, ^22^ ^]^ Wired optical devices have hindered long‐term optogenetic modulation in freely moving animals.

Recent advances in wireless optogenetic technology have overcome this limitation by developing implantable wireless optical devices. Wireless optogenetics enables long‐term in vivo optogenetic control of the central and peripheral nervous system in freely moving animals.^[^ ^23^, ^24^, ^25^, ^26^ ^]^ However, batteries for supplying power to wireless devices would limit storage capacity and lifetime, and increase the weight and size of the devices, which may cause inconveniences during implantation and operation. The development of wireless and battery‐free power supply technologies has provided effective approaches to enable devices with miniaturized size, lightweight, and unlimited operation lifetime.^[^ ^27^ ^]^ The advanced technology of nanogenerators (TENG or piezoelectric nanogenerators) has provided a great chance to convert biomechanical energy in the human body into electricity for powering wireless optical systems.^[^ ^32^, ^33^, ^46^, ^47^, ^48^ ^]^ Thus, in the present study, we developed a wireless self‐powered optogenetic system suitable for wireless cardiac sympathetic modulation in awake freely moving canines. The wireless self‐powered optical system based on a TENG is powered by energy harvested from body motion and realized effective optical illumination that is required for optogenetic neuromodulation. Moreover, we developed a wireless battery‐free implantable optical system with a magnetic resonant coupling external power transmission module that can provide a reliable and constant power supply for long‐term in vivo optogenetic neuromodulation. First, the fully implantable and wirelessly controlled LED device allows for unrestricted animal behavior while performing long‐term optogenetic modulation. Besides, the whole LED implant is battery‐free and wirelessly powered by coupling with the near electromagnetic field of an external wireless charging module, which further minimized the size of the LED implant and extends device operation time. The entire LED implant is encapsulated with soft, biocompatible, and impermeable material to protect the internal electronics. After 4 weeks of implantation, the LED device maintained a normal functional state, ensuring the feasibility of long‐term optogenetic cardiac neuromodulation. Our results showed that long‐term wireless optogenetic modulation using this implantable LED device in freely moving canines significantly inhibited MI‐induced LSG hyperactivity and neural remodeling, suggesting effective long‐term optogenetic cardiac neuromodulation using this wireless LED system.

A major concern for this implantable LED device is the heat generation produced by LED illumination, which might induce alterations in neural activity or damage to surrounding tissue.^[^ ^40^ ^]^ In the present study, we performed in vivo heat test and illustrated that continuous LED illumination increased the temperature of the LED units by around 2 °C, and increased the temperature of the LSG tissue by around 0.5 °C, which is safe and reasonable. Regarding the time point and duration for the daily optogenetic neuromodulation, we took the circadian patterns of the ANS into account. Studies have shown that, in dogs, especially in the post‐MI state, the SG nerve activity showed significant circadian variations, with peak sympathetic activity occurring in the morning.^[^ ^41^, ^49^ ^]^ Our previous study has shown that short‐term sustained LED illumination can induce prolonged LSG neural inhibitory effects, lasting for hours with suppressed LSG neural remodeling. Thus, in the present study, we performed periodic LED illumination (1 h daily in the morning) for optogenetic modulation, to ensure long‐term neural inhibition efficiency while avoiding cumulative heat generation during illumination. Moreover, histological studies showed that no obvious adverse nerve reaction to the 4‐week LED device implantation or illumination was induced in LSG tissue, verifying the long‐term operation safety of the wireless LED system. In addition, the implantation and operation of the LED device did not cause obvious abnormal behavior in animals, indicating that this implantable LED device is suitable and tolerable for the canines. Our results suggest that this wireless implantable LED device enables feasible and effective long‐term optogenetic cardiac neuromodulation in freely moving canines.

### 3.3 Long‐Term Optogenetic Cardiac Neuromodulation Improved Ventricular Electrophysiological Stability and Suppressed Post‐MI VAs

Numerous researches have revealed the key role of sympathetic activation in the generation and maintenance of VAs and SCD, especially when co‐existing with MI‐induced arrhythmogenic substrates.^[^ ^1^, ^2^ ^]^ Simultaneous ECG and LSG neural recording have revealed that post‐MI VAs were preceded by increased sympathetic discharge,^[^ ^2^ ^]^ and LSG activation by electrical stimulation significantly aggravated post‐MI electrophysiological instability and increased malignant VAs.^[^ ^50^, ^51^ ^]^ Therefore, neuromodulation strategies directly targeting the sympathetic nervous system have been utilized to improve post‐MI cardiac remodeling and VAs both in experimental and clinical studies. Left cardiac sympathetic denervation by stellectomy or epidural anesthesia block has been proven efficient to reduce the incidence of VAs in cases of ischemic electrical storms or refractory VAs.^[^ ^4^, ^5^, ^6^, ^7^ ^]^ Our previous study has also proved that optogenetic modulation of cardiac sympathetic nerve activity could increase ventricular electrophysiological stability and prevent acute ischemia‐induced VAs.^[^ ^18^ ^]^ A recent clinical study has shown that stellate ganglion blockade with anesthetic agent injection could reduce the VA burden in patients with VA storms.^[^ ^52^ ^]^ While continuous blockade was associated with greater VA reduction compared with a single injection, which suggests the need for long‐term cardiac sympathetic inhibitory intervention. The present study showed that this implantable wireless optogenetic system enables long‐term precise inhibition of cardiac sympathetic hyperactivity, neural remodeling, and autonomic imbalance (Figures 4 and 5), and thus, improved post‐MI electrophysiological instability with prolonged ERP and APD_90_ and reduced spatial dispersion, resulting in suppressed VA susceptibility (Figure 8).

### 3.4 Long‐Term Wireless Optogenetic Cardiac Neuromodulation Improved Post‐MI Cardiac Remodeling

In the pathological state after MI, myocardial remodeling, such as scar and fibrosis, occurs, leading to electrical heterogeneity and structural remodeling, forming the arrhythmogenic substrate. MI‐induced cardiac sympathetic hyperactivity and neural remodeling could result in heterogeneous sympathetic overflow to the heart, activating the area sympathetic supersensitivity which was located in the noninfarcted myocardium apical to the infarction, thus inducing ERP and APD shortening with increased dispersion and exaggerate electrophysiological instability.^[^ ^53^, ^54^ ^]^ In addition, it could over‐activate adrenergic receptors and promote cardiac microvascular dysfunction, inflammation, and fibrosis, leading to post‐MI cardiac dysfunction and remodeling.^[^ ^55^, ^56^, ^57^, ^58^ ^]^ Previous studies in post‐MI heart failure animal models have shown that selective cardiac afferent denervation prevented excessive cardiac sympathetic activation and improved left ventricular dysfunction and structural remodeling.^[^ ^59^ ^]^ A prospective, randomized clinical trial (NCT 03071653) is underway testing the efficacy of left cardiac sympathetic denervation on VAs, heart failure outcomes, and mortality in heart failure patients.^[^ ^60^ ^]^ In the present study, we also observed multiple cardioprotective effects of long‐term wireless optogenetic cardiac sympathetic inhibition on post‐MI remodeling and VAs. First, long‐term optogenetic modulation with this implantable wireless optogenetic system significantly inhibited MI‐induced LSG hyperactivity and neural remodeling and improved autonomic imbalance, which might reduce the trigger activity and pro‐remodeling effects caused by excessive sympathetic activation. MI‐induced cardiac dysfunction and structural remodeling were significantly improved by long‐term optogenetic cardiac sympathetic inhibition, as manifested by increased LVEF, reduced infarct size, and interstitial fibrosis (Figures 6 and 7). Long‐term optogenetic neuromodulation of cardiac sympathetic ganglion hyperactivity might improve post‐MI remodeling and arrhythmia susceptibility by suppressing sympathetic outflow such as transmitter release to affect the heart via direct neurocardiac synaptic coupling and signaling.^[^ ^61^ ^]^ Overall, our results suggest that long‐term wireless optogenetic inhibition of LSG hyperactivity using this wireless optogenetic system could significantly improve MI‐induced cardiac autonomic, functional, structural, and electrophysiological remodeling, thereby suppressing post‐MI VAs.

### 3.5 Clinical Implications

Our study introduced a wireless self‐powered optogenetic system suitable for wireless cardiac sympathetic modulation in awake freely moving canines. The implantable, battery‐free wireless optical system enabled long‐term precise cardiac neuromodulation, and thus, improved a variety of clinically relevant outcomes in ambulatory post‐MI canines. This innovative self‐powered optical system may provide an opportunity to develop implantable/wearable and self‐controllable devices for long‐term optogenetic therapy. Recently, Mickle and colleagues described a wireless, fully implantable, closed‐loop system for optogenetic neuromodulation, which can sense organ function and trigger responsive optogenetic neuromodulation to precisely restore organ function.^[^ ^16^ ^]^ Studies also proposed a transcutaneous neural recording technology for noninvasive monitoring of skin sympathetic nerve activity (SKNA).^[^ ^62^, ^63^, ^64^ ^]^ Taken together, we can also integrate the cardiac neural monitoring system and the wireless self‐powered optogenetics into a self‐powered closed‐loop system to achieve intelligent optogenetic cardiac neuromodulation for the precise treatment of VAs and other cardiovascular diseases related to sympathetic hyperactivity.

### 3.6 Study Limitation

In the present study, we did not record spontaneous VAs incidence due to the relatively stable feature of the long‐term MI model with rare spontaneous sustained VT or VF, instead, we measured the VA inducibility by programmed electrical stimulation and VF threshold by burst stimulation as surrogates to assess VA susceptibility. In future studies using post‐MI sudden death large animal models prone to spontaneous VA, simultaneous ECG and neural recording might further reveal the accurate and real‐time optogenetic cardiac neuromodulation on arrhythmia termination. For future clinical translation and application, it is also of significance to apply optogenetic sympathetic modulation at different time points after MI or in models of myocardial ischemia‐reperfusion to further validate its cardioprotective effects. In addition, while the present study showed 8‐week stable opsin expression by viral transfection and 4‐week LED device operation which ensured effective optogenetic modulation, several issues, such as the safety of viral vector, long‐lasting opsin expression, and stable optical device operation in humans, need to be solved for further clinical translation and application.

## 4 Conclusion

The wireless self‐powered optical system based on a TENG could realize the effective optical illumination that is required for optogenetic neuromodulation. Long‐term optogenetic neuromodulation significantly improved MI‐induced cardiac autonomic, electrical, functional, and structural remodeling, and thus, suppressed post‐MI ventricular arrhythmogenesis. This innovative self‐powered optical system may provide an opportunity to develop implantable/wearable and self‐controllable devices for long‐term optogenetic therapy.

## 5 Experimental Section

To evaluate the feasibility of this wearable self‐powered optical system, the volunteer fixed the TENG on his elbow joint and tested electrical output, and there was no damage to his human body during the experiment. In addition, all experiments related to the volunteer's elbow joint were conducted in accordance with relevant laws and institutional guidelines, as well as under approval from the Institutional Review Board of Wuhan University. All human subjects gave written and informed consent before participation in the experiments. The animal experiments were approved by the Committee on the Ethics of Animal Experiments of Wuhan University and carried out in accordance with the “Guide for the Care and Use of Laboratory Animals of the National Institutions of Health.” The Supporting Information file contains detailed methods.

## Conflict of Interest

The authors declare no conflict of interest.

## Supporting information

Supporting Information

Supplemental Video 1

## Data Availability

The data that support the findings of this study are available from the corresponding author upon reasonable request.
