# Supplementary material for: Wireless Self‐Powered Optogenetic System for Long‐Term Cardiac Neuromodulation to Improve Post‐MI Cardiac Remodeling and Malignant Arrhythmia
Source: Adv Sci (Weinh). 2023 Jan 25;10(9):2205551. doi: 10.1002/advs.202205551 (PMC10037959; doi:10.1002/advs.202205551)
Supplement: Supplementary file 1 — Supporting Information [file ADVS-10-2205551-s001.pdf]

Supporting Information

Wireless Self-powered Optogenetic System for Long-term Cardiac Neuromodulation to Improve post-MI Cardiac Remodeling and Malignant Arrhythmia

Liping Zhou, MD, Yuanzheng Zhang, PhD, Gang Cao, PhD, Chi Zhang, PhD, Chen Zheng, BE, Guannan Meng, MD, Yanqiu Lai, MD, Zhen Zhou, MD, Zhihao Liu, MD, Zihan Liu, MD, Fuding Guo, MD, Xin Dong, MA, Zhizhuo Liang, BE, Yueyi Wang, PhD, Shishang Guo, PhD, Xiaoya Zhou, MD, PhD, Hong Jiang, MD, PhD*, Lilei Yu, MD, PhD**

Supplemental Material List:

Materials and Methods (Page 2-8)
Figure S1. Dependence of the output current at different external load resistances.**(Page 9)**
Figure S2. The wireless LED system design and circuit diagram. **(Page 10)**
Figure S3. Thermal property measurement on the surface of LED and the surface of the LSG during 1h illumination. **(Page 11-12)**
Figure S4. Serum inflammatory markers of IL-1β, IL-6 and TNF-α at baseline and 4-week after optogenetic neuromodulation. **(Page 13)**
Figure S5. Representative images of HE staining of LSG from four groups and neuronal diameter quantitative results displayed as ratio data. **(Page 14)**
Figure S6. Longitudinal data of BP and HR during the experiment. **(Page 15)**
Figure S7. MI staining images and quantification. **(Page 16)**
Figure S8. Diagram showing the epicardial locations of ERP and APD measurement sites and the infarct zone location. **(Page 17)**
Movie S1. Movie of the wireless LED system working mode. **(Page 18)**

Materials and Methods

Experimental Material

Gola particle (Au, 99.9%) and chromium (Cr, 99%) for preparing electrodes were purchased from Goodwillmetal. Co. Ltd. VBH tap was purchased for 3M Co. Ltd. Polyethylene terephthalate (PET) film from the Alibaba online store and used as the friction material.

Fabrication of flexible TENG

The Au/Cr/PET (50 nm/200 µm) multilayer structure has been prepared by thermal evaporation (JSD-350 thermal evaporator). Cr (10 nm) is used as a binder to increase the adhesion of the gold to the substrate during the evaporation process. Then, two Au/Cr/PET multilayer structures are then glued together with VBH tape to form a gap (95×13×3 mm, L×W×H) in the middle. By now, a TENG based on vertical contact-separation mode has been accomplished.

Measurements and characterization

Scanning electron microscopy (Tescan SOLARIS) was employed to characterize the surface morphology of the samples. The resistance of the electrode under different bending angles by a multimeter (UT39A, UNI-T, China). The output voltage and current were measured by an oscilloscope (Tektronix TDS2012B, USA) and an electrometer (Keithley 6514). The charging curves of the capacitor were recorded by an electrochemistry workstation (CHI 660E).

Construction of wireless LED system

The wireless LED system is composed of the internal LED implant and the external powering module (**Figure 1b**). The wireless LED implant consists of two main parts. The first part is the power receiver consisting of a six-turn coil and a rectifier. The coil extracts Radio-Frequency (RF) energy coupled from the external module and the rectifier converts the RF energy into current with fixed frequency at 20 Hz and duty at 40% to make LED work periodically. The second part is the light-delivery portion, routing the direct current to the 6-unit-coupled LED (wavelength 565 nm). For overall encapsulation, a 1 mm of soft silicone rubber sheet material is applied to the entire exterior of the device to form a biocompatible and impermeable coating to protect the internal electronics. The overall device dimensions are 45mm×40mm×3mm (L×W×thickness) of the power receiver part and 80mm×8mm×3mm (L×W×thickness) of the light-delivery part.

As to the external powering module, an inverter first converted the direct current provided by a 7.5V battery into alternating current, which was used to supply another six-turn coil to create an alternating electric field, coupling with the internal coil to transmit the RF energy. The external powering module was wireless and has dimensions of 80mm×80mm×10mm

(L×W×thickness). For LED illumination, the external module could be fixed at the corresponding position on the chest wall to power the internal implanted LED device.

Animal preparation

The animal study was approved by the Committee on the Ethnics of Animal Experiments of the Wuhan University and carried out in accordance with the “Guide for the Care and Use of Laboratory Animals of the National Institutions of Health”. Twenty-four male beagle canines weighing 9 to 11 kg were supplied by the experimental animal center in medical school of Wuhan University. All surgeries were performed under pentobarbital sodium anesthesia (30mg/kg, i.v. for induction, followed by 60mg/h for maintainance) with mechanical ventilation (MAO01746, Harvard Apparatus Holliston, Massachusetts). All efforts were made to minimize suffering.

Study protocol

The study protocol was outlined in **Figure 4a**. Twenty-four beagle canines were randomly divided into the control group (n=6, AAV2/9-hsyn-GFP-WPRE-pA injection with sham MI), the ArchT group (n=6, AAV2/9-hsyn-ArchT-GFP-WPRE-pA injection with MI), the LED group (n=6, AAV2/9-hsyn-GFP-WPRE-pA injection with MI and LED illumination), and the optogenetic neuromodulation (ON) group (n=6, AAV2/9-hsyn-ArchT-GFP-WPRE-pA injection with MI and LED illumination). Four weeks after AAV microinjection to transfect genes encoding an inhibitory opsin ArchT and/or tag protein GFP into LSG neurons, the wireless LED device was implanted on top of the LSG to perform illumination (565nm) for optogenetic modulation. MI was induced by left anterior descending occlusion (LADO) and then LED illumination was performed for 1h immediately after MI and daily in the morning for four weeks. After 4-week neuromodulation, canines were anesthetized with mechanical ventilation. Transthoracic echocardiography was performed to assess cardiac function, and ECG was recorded for HRV analysis. Then the thoracotomy was performed to expose the LSG and heart. LSG function and neural activity, as well as the electrophysiological studies of ERP, APD, and arrhythmia inducibility were measured. At the end of the experiment, the LSG and heart tissue were harvested for histological staining.

Viral injection into LSG

The virus AAV2/9-hsyn-ArchT-GFP-WPRE-pA was chosen to transfect LSG. A similar construct (AAV2/9-hsyn-GFP-WPRE-pA) without ArchT was used as a control. The virus was purchased from BrainVTA Co. Ltd. (Wuhan, China). After anesthesia, a left thoracotomy was performed at the third intercostal space and the LSG was carefully exposed. Virus solution (AAV2/9-hsyn-ArchT-GFP-WPRE-pA: 30μl, 5.4×10^{12} vector genomes/ml;

AAV2/9-hsyn-GFP-WPRE-pA: 30µl, 5.0×10^{12} vector genomes/ml) were injected at 1 µl/min into four sites, using a 25 µl Hamilton syringe connected to a Harvard PHD syringe pump (Harvard Apparatus, Holliston, MA, USA). After the injection, the chest was closed in layers and the pneumothorax was reduced. Antibiotics were given during and 3 days after surgery.

Thermal test of the LED device

We characterized the thermal property of the implanted LED device in vivo using an optical fiber temperature sensor system. The fiber-optics sensor was placed on the surface of the LED units, together inside the encapsulated soft silicone rubber sheet coating, and then implanted onto the surface of the LSG, to measure the thermal effect of LED illumination. In view of the insulation property of the encapsulation, we also measured the temperature on the surface of LSG where is outside of the encapsulated soft silicone rubber sheet coating, to detect the thermal response on the LSG tissue.

LED device implantation and location identification

Four weeks after viral injection, the canines were anesthetized and a left-sided thoracotomy was conducted at the third intercostal space to expose the LSG. The 6-unit-coupled monochromatic LED (wavelength 565 nm) was implanted onto the LSG to illuminate LSG for optogenetic neuromodulation. The power receiver connecting the LED was inserted between the chest skin and muscle to receive wireless electromagnetic power supply and control the LED. (**Figure 3b**) The external powering module was placed on the corresponding position of chest for wireless power supply. The LSG was illuminated by consecutive flashes (20Hz, 40% duty cycle, 20ms pulse width, 5mW/mm^2). LED illumination was performed 1 h daily for four weeks. Four weeks after implantation, the position of implanted LED devices was illustrated by X-ray radiation (**Figure 3c**).

Myocardial infarction model

After thoracotomy, the pericardium was carefully opened to expose the heart. The MI was induced by completely ligating the left anterior descending coronary artery (LAD) below its first diagonal branch without reperfusion. ECG during the first hour of MI was recorded for 1h, and defibrillation was performed if ventricular fibrillation occurs during observation. After 1h of observation, the chest was closed in layers.

Echocardiography

Transthoracic echocardiography was performed 4 weeks after MI using a VividE9 system (GE Vingmed Ultrasound As, Horten, Norway) with dogs under anesthesia with 30mg/kg pentobarbital sodium. LV end-diastolic volume (LVEDV), end-systolic volume (LVESV) and

ejection fraction (LVEF) were measured and calculated by biplane Simpson's method. The peak early (E) and late (A) filling velocity were measured and the ratio of E to A (E/A) was calculated. LV wall thickness (WT) at end-systole and end-diastole were measured at the apex, LV posterior wall (LVPW) and intraventricular septal (IVS), and % systolic wall thickening (%WT) was calculated as 100×(systolic wall thickness - diastolic wall thickness)/diastolic wall thickness.

Tissue Doppler imaging with low-dose dobutamine stress (10ug/kg/min) was performed to evaluate LV regional wall-motion abnormality and myocardial functional reserve. According to the 17-segment model of AHA guidelines [1], the LAD branch perfuses 7 ventricular segments: 1(basal anterior), 2(basal anteroseptal), 7(mid anterior), 8(mid anteroseptal), 13(apical anterior), 14(apical septal), and 17(apex) (**Figure 6i**). The peak systolic velocity of 7 LAD-perfused segments was measured by tissue Doppler imaging at rest and during low-dose dobutamine stress. The average peak systolic velocity of 7 segments were calculated. All parameters were evaluated blind to the intervention by the same experienced echocardiographer.

Measurement of HRV

ECG was recorded using the PowerLab data acquisition system (4/35, AD Instruments, New South Wales, Australia). Five-minute ECG segments were used for HRV analysis with Labchart 8 software. The following power spectral variables were determined: the very low-frequency component (VLF, 0-0.04Hz), the low-frequency component (LF, 0.04-0.15Hz) and the high-frequency component (HF, 0.15-0.4Hz). The normalized unit of LF and HF (LFnu and HFnu) and the ratio between LF and HF (LF/HF) were calculated.

Measurement of LSG function and neural activity

LSG function was assessed by the percentage of maximal blood pressure (BP) increase induced by high-frequency stimulation (20Hz, 0.1ms pulse duration) at 5 incremental levels (level 1=1 to 4 V; level 2=5 to 7 V; level 3=7.5 to 10 V; level 4=10 to12.5 V; level 5=12.5-15V) as descried previously[2]. Each high-frequency stimulation lasted < 30 seconds, and the later measurement was not taken until BP returned to a normal level. Neural activity from the LSG was recorded using a Power Lab data acquisition system (4/35, AD Instruments, New South Wales, Australia). A bipolar tungsten-coated micro-electrode was inserted into the LSG, and a ground lead was connected to the chest wall. The electrical signals generated by the LSG were recorded and analyzed for 1 minute. Neural activity, characterized by the recorded amplitude and frequency, was defined as deflections with a signal-to-noise ratio 3:1 and determined as described previously.[3]

Measurement of ventricular effective refractory period (ERP)

Ventricular elecrtrophysiological properties were measured epicardially. Under anesthesia and mechanical ventilation, bilateral thoracotomy was performed to expose the heart. As shown in the **Figure S8**, bipolar electrode catheters were sutured at the following 6 epicardial sites of the ventricle to measure the ERP and APD: the LV apical peri-infarct zone (LVA), the LV median area (LVM), the LV base (LVB), the right ventricular apex (RVA), the RV median area (RVM) and the RV base (RVM). The ventricular ERP at each site was determined by programmed pacing with 8 consecutive stimuli (S1–S1, 330-ms cycle length) followed by a premature stimulus (S2). The S1–S2 interval was progressively shortened until refractoriness was achieved. ERP was defined as the longest S1–S2 interval that failed to capture the ventricles. The spatial dispersion of ERP was defined as coefficient of variation [(standard deviation/mean)×100%] of ERP among 6 sites.

Measurement of ventricular action potential duration (APD)

A dynamic steady-state pacing (S1S1) was performed to record the epicardial monophasic action potential (MAP) from 6 sites of the left and right ventricle using a custom-made Ag–AgCl catheter. The pulse train at the cycle length of 330ms was delivered and maintained for 30s to ensure a steady state. A 2-min interruption was then taken to minimize the pacing memory effects. The MAPs were recorded by the LEAD 7000 workstation system (Lead 7000B, Jinjiang Inc. China). The APD_{90} was measured at the 90% repolarization. The spatial dispersion of APD_{90} was determined by calculating the coefficient of variation of APD_{90} among 6 sites.

VA inducibility and VF threshold measurement

VA inducibility and VF threshold were measured epicardially, via a bipolar electrode catheter sutured on the epicardium of the right ventricular apex. Programmed electrical stimulation was performed at twice the diastolic threshold with 8 S1-S1 consecutive stimuli (330ms cycle length) followed by extra-stimulus (S2-S3-S4). Initial premature extra-stimulus (S2) was conducted with S1-S2 interval 30 ms longer than the ERP and progressively shortened by 10ms decrements until VT/VF was induced or refractoriness was achieved. If ventricular tachycardia (VT) or VF were not induced, a second extra-stimulus (S3) and then a third extra-stimulus (S4) was delivered until refractoriness was achieved. VA, including VT and VF, was considered sustained when it lasted >15 beats. As previously described [4], VA inducibility was scored as follows: 0, non-inducible with three extrastimuli; 1, non-sustained VT induced with three extrastimuli; 2, sustained VT or VF induced with three extrastimuli; 3, non-sustained VT induced with two extrastimuli; 4, sustained VT or VF induced with two

extrastimuli; 5, non-sustained VT induced with one extrastimulus; 6, sustained VT or VF induced with one extrastimuli; and 7, VT/VF induced during the eight pacing beats. When multiple forms of arrhythmia occurred in 1 heart, the highest score was recorded. Defibrillation was performed if VF was induced and a 20min rest period was taken before VF threshold measurement. Burst pacing (20Hz, 0.1ms pulse duration, 10s) was supplied to the right ventricular apex to measure VF threshold. The pacing voltage started at 2V and by 2V increments until VF was induced. The lowest voltage that induced VF was defined as the VF threshold.

Myocardial infarct size determination

The myocardial infarct size was assessed with triphenyltetrazolium chloride (TTC) staining in dogs of optogenetics group and MI group. The myocardial infarct size measurement was performed by the same experienced experimental technician who was blinded to the intervention. At the end of the experiment, the heart was quickly removed and frozen at −20°C for 3 hours and then was cut into 5-6 slices (5-10 mm thick) from apex perpendicularly to the LAD occlusion site. Slices were incubated in 1.0% triphenyltetrazolium chloride at 37°C for 15 minutes to discriminate the infarct tissues from the viable myocardium. After overnight fixation with 4% paraformaldehyde, each slice was photographed with a digital camera. The infarct area and the LV area were determined using Image-Pro Plus software version 6.0 (MediaCybernetics, Carlsbad, CA, USA). Myocardial infarct size was calculated as the ratio of the infarct area to the total LV area.

Histopathological staining

At the end of the experiment, the LSG and the basal LV tissue above LAD occlusion site were harvested for histopathological staining. Tissues were fixed in 4% paraformaldehyde, embedded in paraffin and sectioned into 4 μm slices. HE staining was performed to observe tissue structure and neuron size in LSG tissue. Double immunofluorescence staining was used for tyrosine hydroxylase (TH) (Abcam, Cambridge, England, or Servicebio, China) with GFP (Abcam, Cambridge, England) to assess GFP-ArchT transfection in LSG, with c-Fos and NGF (Servicebio, China) to assess the LSG remodeling. The nuclei were stained with 4, 6-diamidino-2-phenylindole (DAPI). Masson’s trichrome staining was performed for LV fibrosis analysis. Anti-TH (Lifespan Biosciences) imunohistochemical staining was performed to assess myocardial sympathetic nerve fiber innervation. The fibrosis and TH expression was quantified using the Image-Pro Plus 6.0 (MediaCybernetics, Carlsbad, CA, USA).

Measurement of plasma norepinehrine and neuropeptide Y concentration

Blood samples from jugular vein were obtained 4 weeks after MI. Plasma was collected and stored at -80°C. The norepinehrine (NE) and neuropeptide Y (NPY) concentrations were measured with the enzyme-linked immunosorbent assay kit (NE, IBL international, Tecan Trading AG, Switzerland; NPY, EZHNPY-25K, Sigma-Aldrich, St. Louis, MO, United States).

Statistical analysis

All continuous variables were presented as the mean ± standard deviation (SEM) and tested for a normal distribution using the Shapiro-Wilk normality test. The continuous variables were analyzed by one-way analysis of variance or two-way repeated-measures analysis of variance followed by Tukey's multiple comparisons test and P values were adjusted. All data were analyzed using the GraphPad Prism software (version 8.0, GraphPad Software Inc., San Diego, CA). All statistical analyses were two-sided, and $P < 0.05$ was considered statistically significant.

References

1. Cerqueira MD, Weissman NJ, Dilsizian V, Jacobs AK, Kaul S, Laskey WK, Pennell DJ, Rumberger JA, Ryan T, Verani MS; American Heart Association Writing Group on Myocardial Segmentation and Registration for Cardiac Imaging. Standardized myocardial segmentation and nomenclature for tomographic imaging of the heart. A statement for healthcare professionals from the Cardiac Imaging Committee of the Council on Clinical Cardiology of the American Heart Association. Circulation. 2002 Jan 29;105(4):539-42.

2. Yu L, Zhou L, Cao G, Po SS, Huang B, Zhou X, Wang M, Yuan S, Wang Z, Wang S, Jiang H. Optogenetic Modulation of Cardiac Sympathetic Nerve Activity to Prevent Ventricular Arrhythmias. J Am Coll Cardiol. 2017 Dec 5;70(22):2778-2790.

3. Yu L, Scherlag BJ, Li S, Fan Y, Dyer J, Male S, Varma V, Sha Y, Stavrakis S, Po SS. Low-level transcutaneous electrical stimulation of the auricular branch of the vagus nerve: a noninvasive approach to treat the initial phase of atrial fibrillation. Heart Rhythm. 2013 Mar;10(3):428-35.

4. Yu L, Wang S, Zhou X, Wang Z, Huang B, Liao K, Saren G, Chen M, Po SS, Jiang H. Chronic Intermittent Low-Level Stimulation of Tragus Reduces Cardiac Autonomic Remodeling and Ventricular Arrhythmia Inducibility in a Post-Infarction Canine Model. JACC Clin Electrophysiol. 2016 Jun;2(3):330-339.

Supplementary Figures

Figure S1

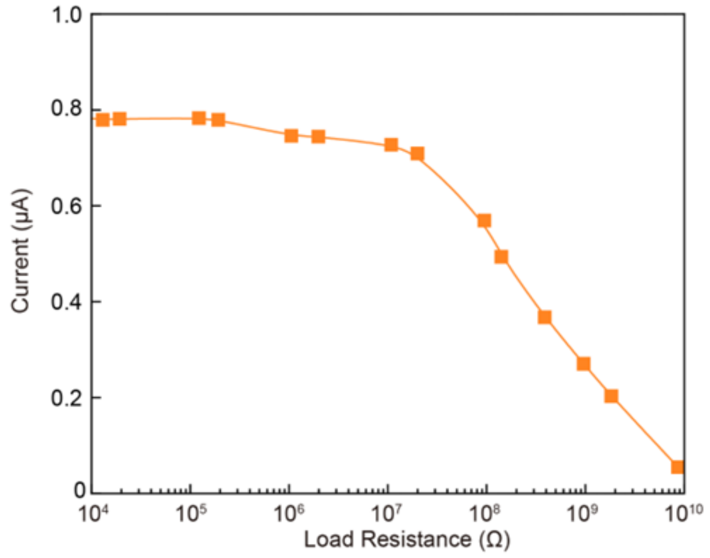

Figure S1. Dependence of the output current at different external load resistances.

Figure S2

(a)

(b)

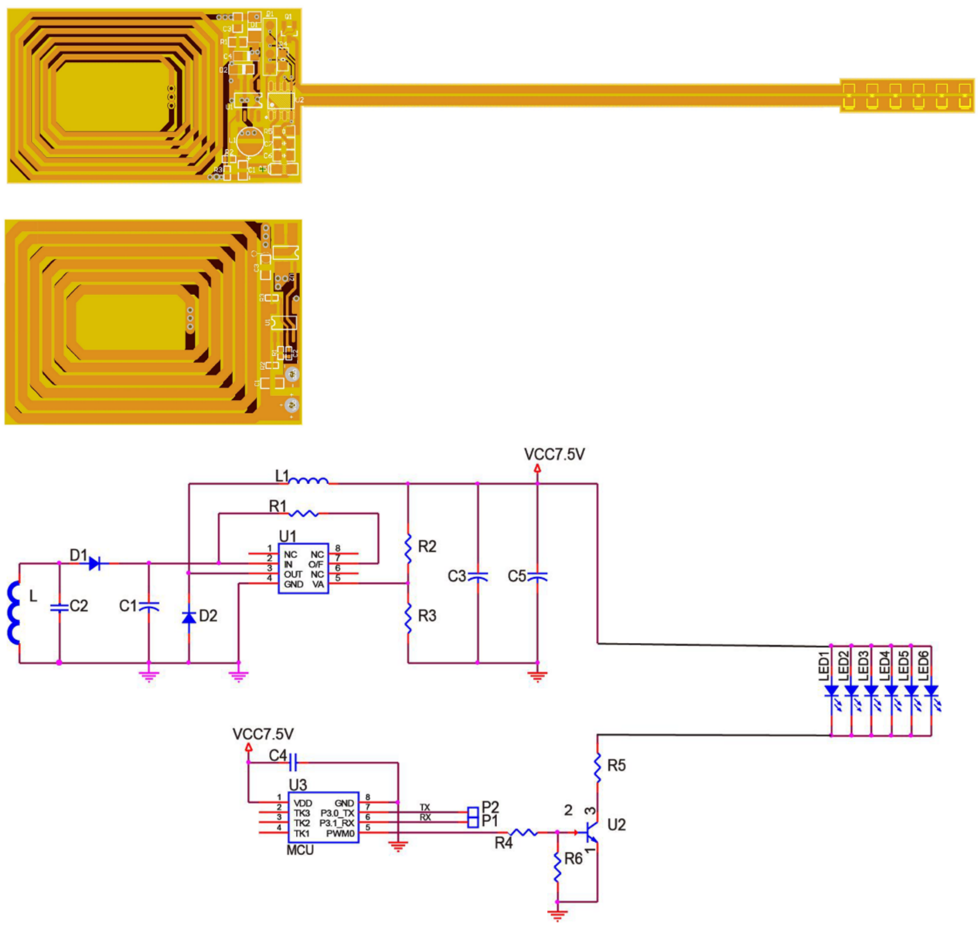

Figure S2. The wireless LED system design and circuit diagram. The detailed system design (**a**) and circuit (**b**) diagram of the LED system.

Figure S3

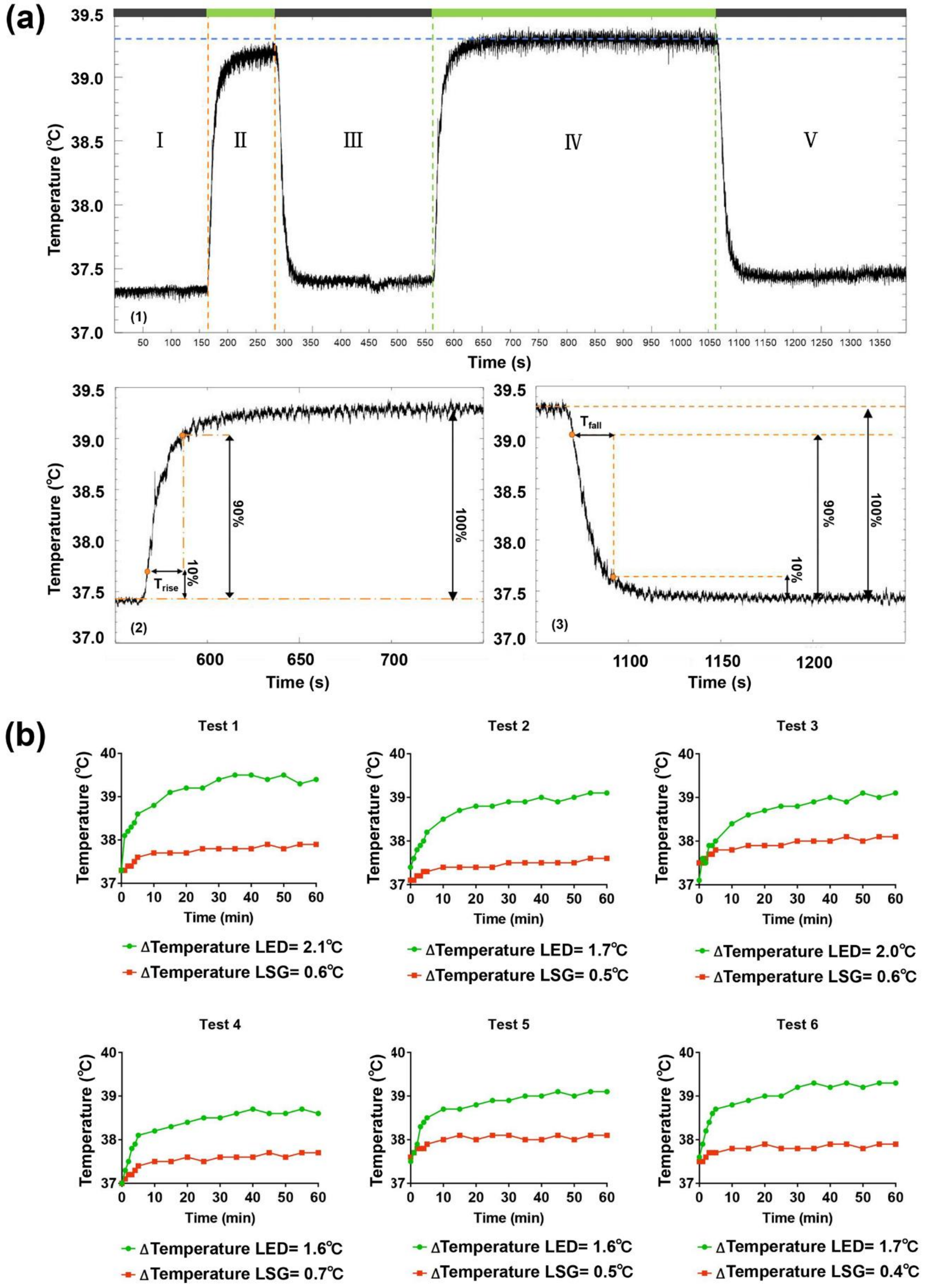

Figure S3. Thermal property measurement on the surface of LED and the surface of the LSG during 1h illumination. (**a**)Temperature-to-time curve at different conditions on the surface of LED. **Part I:** 0 to 160 sec, LED is off. The measured temperature is the normal

body temperature, with the value of 37.3℃, which is consistent with the animal normal body temperature. **Part II:** 160 sec to 280 sec, LED is on. The temperature rises up fast in few seconds and then slowly reaches a stable level around 39.2℃ and then becomes steady. **Part III:** 280 sec to 560 sec, LED is off. The temperature falls quickly to the same level at baseline (37.4℃). **Part IV:** 560 sec to 1560 sec, LED is on. With prolonged LED illumination, the temperature increases the same with part II and then reaches a stable value at around 39.3℃. **Part V:** 1560 sec to 1450 sec, LED is off. The temperature goes down at around 37.4℃. (**b**) Thermal property measurement on the surface of LED and the surface of the LSG during 1h illumination.

Figure S4

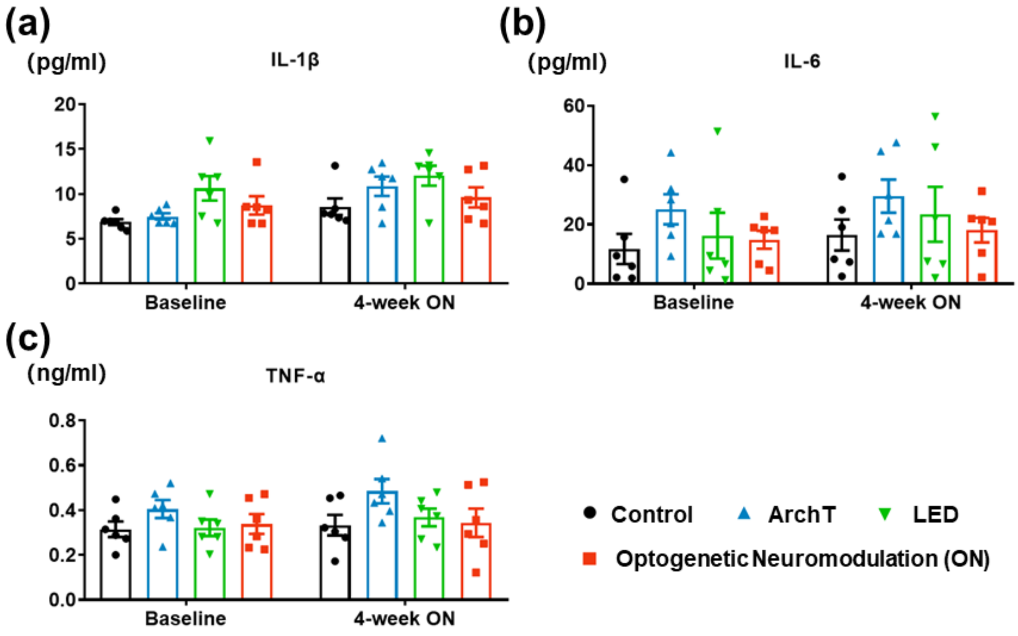

Figure S4. Serum levels of inflammatory cytokines of IL-1β, IL-6 and TNF-α at baseline and 4-week after optogenetic neuromodulation.

Figure S5

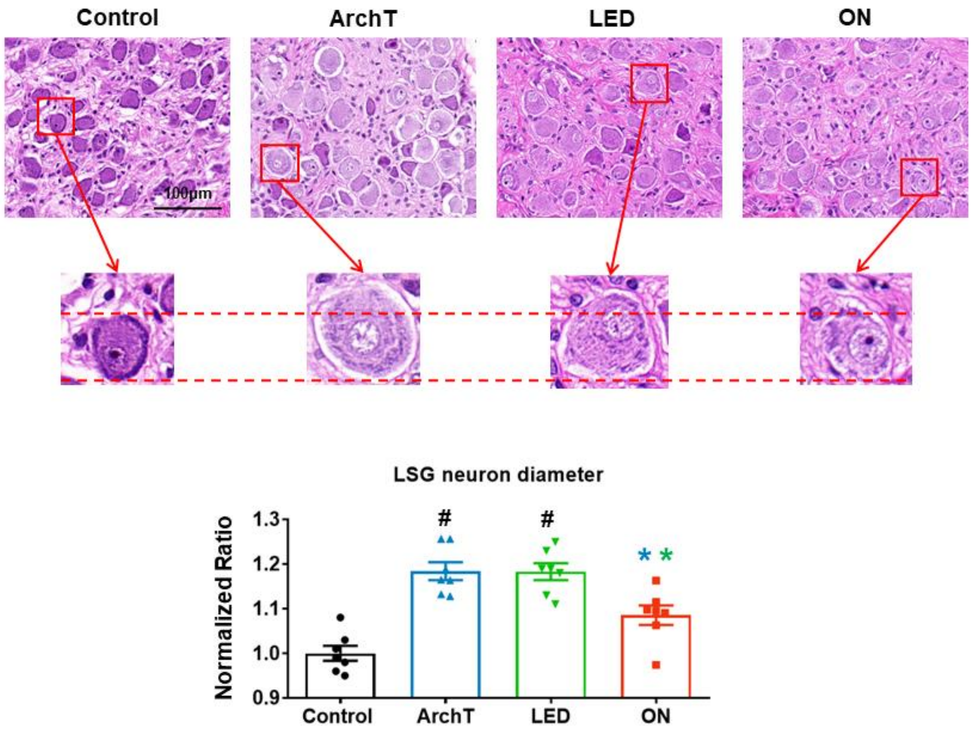

Figure S5. Representative images of HE staining of LSG from four groups and neuronal diameter quantitative results displayed as ratio data.

Figure S6

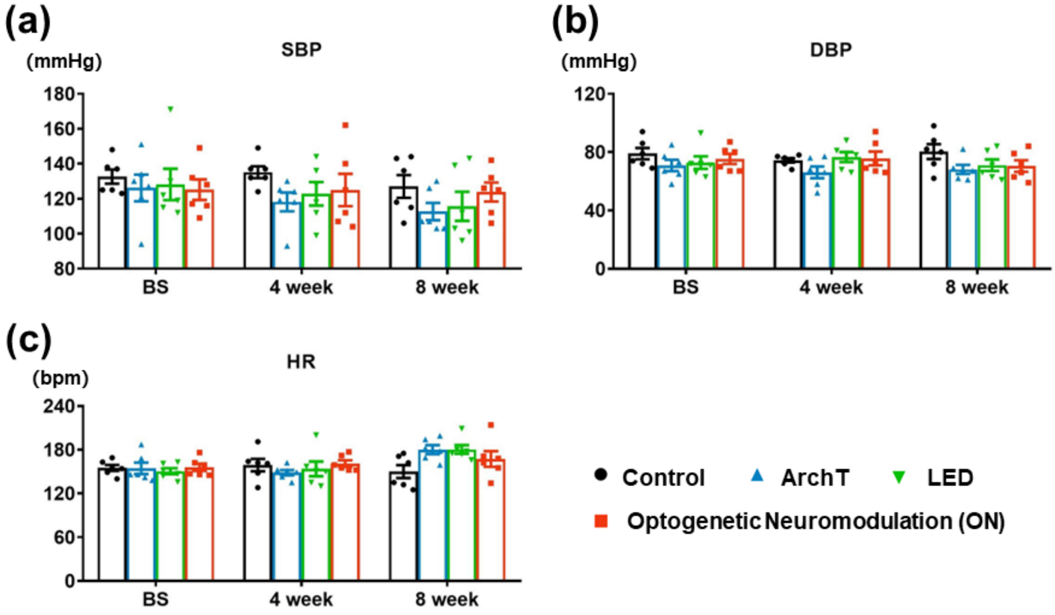

Figure S6. Longitudinal data of BP and HR during the experiment. Systolic and diastolic BP (**a, b**) and HR (**c**) at baseline, 4 weeks and 8 weeks after AAV injection.

Figure S7

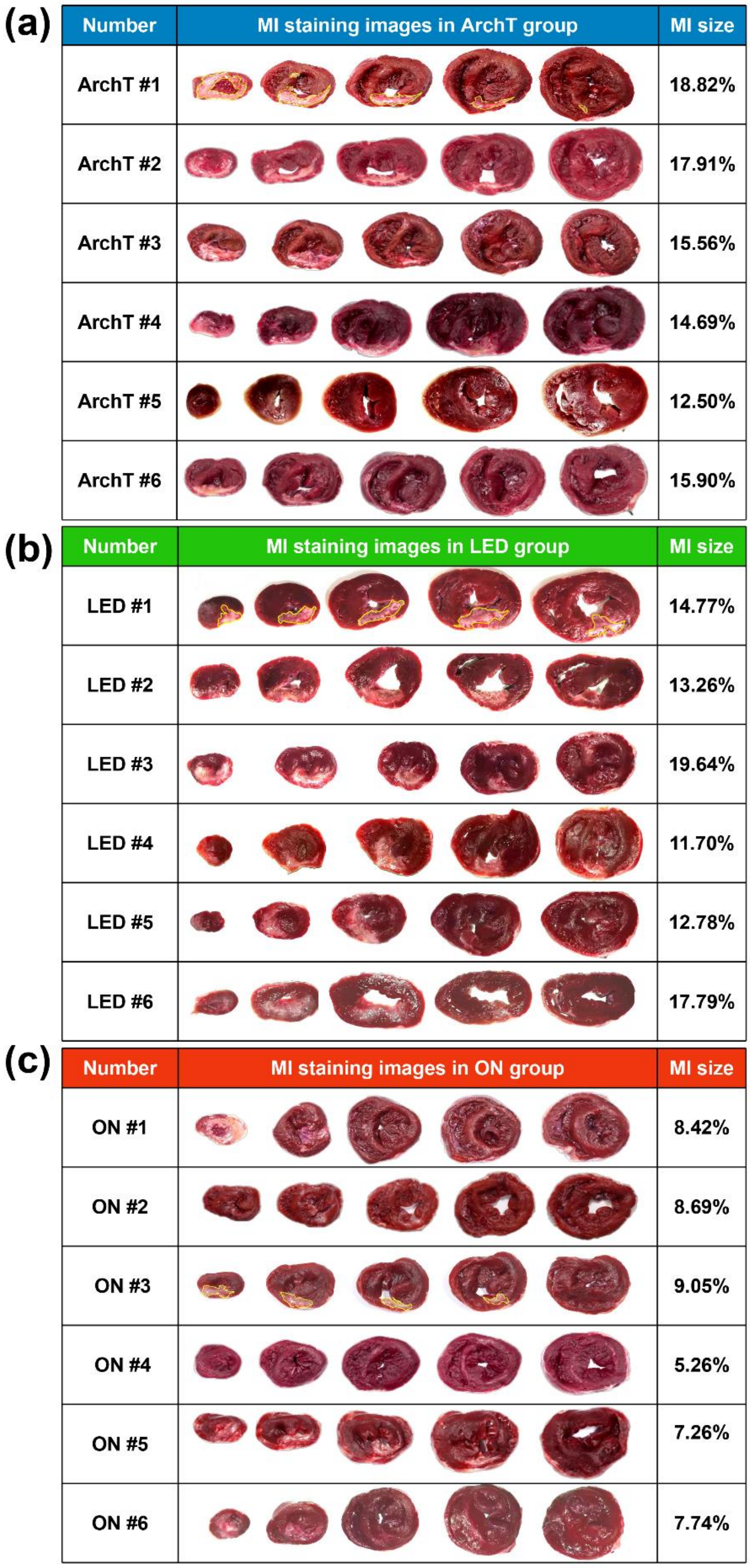

Figure S7. MI staining images and quantification. MI staining images in the ArchT group (**a**), the LED group (**b**) and the ON group (**c**), and MI size quantification.

Figure S8

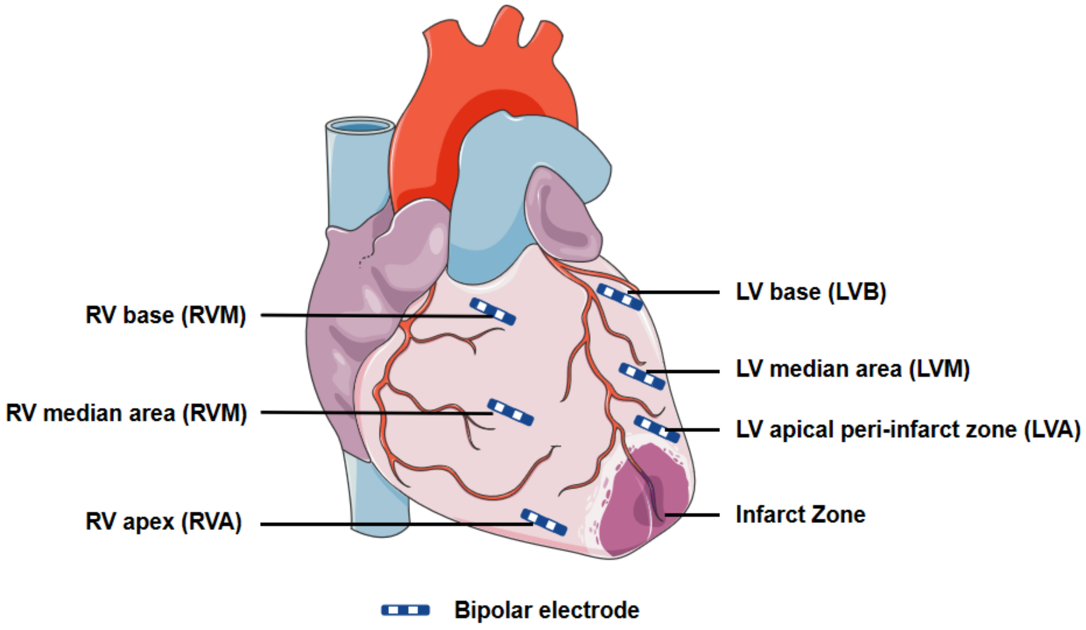

Figure S8. Diagram showing the epicardial locations of ERP and APD measurement sites and the infarct zone location.

Movie S1. Movie of the wireless LED system working mode. The wireless LED system illuminates at 20 Hz frequency, 40% duty cycle and 565nm wavelength.
